# A national study of the molecular epidemiology of HIV-1 in Australia 2005–2012

**DOI:** 10.1371/journal.pone.0170601

**Published:** 2017-05-10

**Authors:** Alison Castley, Shailendra Sawleshwarkar, Rick Varma, Belinda Herring, Kiran Thapa, Dominic Dwyer, Doris Chibo, Nam Nguyen, Karen Hawke, Rodney Ratcliff, Roger Garsia, Anthony Kelleher, David Nolan, The Australian Molecular Epidemiology Network-HIV (AMEN-HIV)

**Affiliations:** 1 Department of Clinical Immunology, Royal Perth Hospital, Perth Western Australia; 2 Western Sydney Sexual Health Centre, Western Sydney Local Health District, Parramatta NSW Australia; 3 Marie Bashir Institute for Infectious Diseases and Biosecurity, University of Sydney, NSW,Australia; 4 Centre for Infectious Diseases and Microbiology Laboratory Services, ICPMR-Pathology West (NSW Health Pathology), Westmead Hospital and University of Sydney, Westmead NSW, Australia; 5 HIV Characterisation Laboratory, Victorian Infectious Diseases Reference Laboratory, Doherty Institute, Melbourne, Victoria, Australia; 6 Division of Immunology, HSQ Pathology Queensland Central Laboratory, RBWH Herston, Queensland, Australia; 7 South Australian Health and Medical Research Institute, Adelaide, South Australia; 8 School of Medicine, Flinders University, Adelaide, South Australia; 9 Clinic 275, Royal Adelaide Hospital, Adelaide, South Australia; 10 Department of Microbiology and Infectious Diseases, SA Pathology, Adelaide South Australia; 11 The Clinical Immunology Department, Royal Prince Alfred Hospital, Sydney NSW, Australia; 12 The Kirby Institute for Infection and Immunity in Society, University of New South Wales, Sydney NSW Australia; Institut Pasteur of Shanghai Chinese Academy of Sciences, CHINA

## Abstract

**Introduction:**

Rates of new HIV-1 diagnoses are increasing in Australia, with evidence of an increasing proportion of non-B HIV-1 subtypes reflecting a growing impact of migration and travel. The present study aims to define HIV-1 subtype diversity patterns and investigate possible HIV-1 transmission networks within Australia.

**Methods:**

The Australian Molecular Epidemiology Network (AMEN) HIV collaborating sites in Western Australia, South Australia, Victoria, Queensland and western Sydney (New South Wales), provided baseline HIV-1 partial *pol* sequence, age and gender information for 4,873 patients who had genotypes performed during 2005–2012. HIV-1 phylogenetic analyses utilised MEGA V6, with a stringent classification of transmission pairs or clusters (bootstrap ≥98%, genetic distance ≤1.5% from at least one other sequence in the cluster).

**Results:**

HIV-1 subtype B represented 74.5% of the 4,873 sequences (WA 59%, SA 68.4%, w-Syd 73.8%, Vic 75.6%, Qld 82.1%), with similar proportion of transmission pairs and clusters found in the B and non-B cohorts (23% vs 24.5% of sequences, *p* = 0.3). Significantly more subtype B clusters were comprised of ≥3 sequences compared with non-B clusters (45.0% vs 24.0%, *p* = 0.021) and significantly more subtype B pairs and clusters were male-only (88% compared to 53% CRF01_AE and 17% subtype C clusters). Factors associated with being in a cluster of any size included; being sequenced in a more recent time period (p<0.001), being younger (*p*<0.001), being male (*p* = 0.023) and having a B subtype (*p* = 0.02). Being in a larger cluster (>3) was associated with being sequenced in a more recent time period (*p* = 0.05) and being male (*p* = 0.008).

**Conclusion:**

This nationwide HIV-1 study of 4,873 patient sequences highlights the increased diversity of HIV-1 subtypes within the Australian epidemic, as well as differences in transmission networks associated with these HIV-1 subtypes. These findings provide epidemiological insights not readily available using standard surveillance methods and can inform the development of effective public health strategies in the current paradigm of HIV prevention in Australia.

## Introduction

HIV-1 is highly genetically variable with a continual rapid mutation and recombination associated with an error-prone and non-proofreading reverse transcriptase activity [1]. There are four distinct HIV-1 groups (M, N, O and P) of which the M group accounts for 90% of infections worldwide. Within the M group there are nine phylogenetically distinct subtypes (A-D, F-H, J and K) along with an increasing number of inter-subtype circulating recombinant forms (CRFs). The main HIV-1 subtypes have distinct geographical associations that can provide useful epidemiological information [2], although there is growing evidence of increasing subtype and inter-subtype HIV-1 genetic diversity in regions previously characterised by specific HIV-1 subtypes [3–6]. Globally, subtype C is the most prevalent and is strongly associated with sub-Saharan African and Indian populations, followed by subtype A (east Africa) and subtype B (western Europe, United States and Australia); these jointly account for 70% of HIV infections [2,7]. Other major subtypes (F, H, J and K) have remained stable at low levels, accounting for around 1% of infections worldwide, whilst subtype D has decreased over time [2]. HIV-1 CRFs account for around 17% of infections worldwide; a 50% increase in the number of total global HIV-1 infections between 2000 and 2007. Unique recombinant forms (URFs) account for approximately 4% of all HIV infections globally, though this proportion can increase to as high as 30% of all new infections in regions where multiple subtypes and CRFs co-circulate, known as recombinant hotspots [8,9].

Although HIV-1 subtype diversity exists in Africa where HIV-1 infection has long been established [2,10,11], recent evidence shows previously geographically-restricted HIV-1 subtypes and CRFs have now migrated to broader regions of the world [2,4–6,12,13]. This increasing global HIV-1 diversity may have important clinical implications given that HIV-1 subtypes have been associated with differences in disease progression [14–16], transmissibility [17,18] susceptibility to antiretroviral therapy [14,19], HIV-1-specific immune responses relevant to *in vivo* infection and vaccine design [20], as well as risk of age-related diseases [21]. Importantly, increased HIV-1 subtype diversity including inter-subtype recombinant forms pose a challenge in HIV diagnostic laboratories, particularly pertaining to HIV-1 RNA assays where the accuracy of results may be influenced by non-B subtype sequence variation [22].

Australia has a history of strong community engagement, effective public health and clinical management strategies that have contributed to a low national HIV-1 prevalence of ~158/100,000 population, with ~26,800 people currently living with HIV [23]. Despite this, the number of new diagnoses has increased by 26% since 2003: this includes 1,236 new cases in 2014, representing a 10% increase over the numbers diagnosed in 2011 [23]. This is set against an overall downward global trend in new HIV-1 diagnoses, most notably in sub-Saharan Africa and also evident in the Asia Pacific region [24].

The Australian HIV epidemic has previously been characterised by a high prevalence of HIV-1 subtype B infection across all risk categories [25–27]. Recently however, there has been a reported increase of imported HIV-1 infection via migration or overseas travel [28] to and from areas where known HIV-1 diversity has been established or from areas where HIV-1 infections are increasing or highly prevalent [24,29,30] and among risk groups other than men who have sex with men (MSM) [29]. Given the ongoing rise in new HIV infections in Australia, investigating HIV-1 subtype distribution may provide valuable information that can inform prevention strategies, while also ensuring that laboratory monitoring is appropriate for the local epidemic. This concept is supported by recent epidemiological studies of HIV-1 sequences that have shown marked increases in the prevalence of non-B subtypes and CRFs [3,4,12] and previously described in previous Australian studies [31–33].

Sequence analysis of the HIV-1 *pol* region, used for detecting antiretroviral drug resistance, can also be utilised for subtype determination and phylogenetic analysis. These analyses can be used to monitor sequence similarities, follow the introduction of new subtypes, and have increasingly been utilised to characterise transmission clusters and networks in order to trace the global diversity of HIV-1 [34,36,37] and how this has changed over time [38–40] and within different risk groups [41,42]. This methodology has been applied on a global scale to study transmission networks [34] and to investigate the role of travel in the spread of HIV-1 within Europe [6,39], the United States [38] and the United Kingdom [41], therefore providing a better understanding of sequence dynamics to assist real time HIV-1 surveillance and potentially prevent further HIV infections.

In order to provide a national population-based platform for these analyses, we have established a collaborative network of all Australian HIV-1 sequencing laboratories, which together provide accredited HIV-1 sequencing for clinical management throughout all Australian states and territories. This network has been identified as the Australian Molecular Epidemiology Network (AMEN). We have performed a retrospective analysis of predominantly baseline (pre-treatment) HIV-1 sequences to determine HIV-1 subtype distribution and phylogenetic structure within Australia during the period 2005–2012, with the aim of supporting rational, evidence-based approaches to prevent, treat and monitor HIV-1 infection within Australia and its linked transmission networks.

## Materials and methods

### Sample population and dataset collation

This study was conducted within the framework of the Australian Molecular Epidemiology Network (AMEN), a collaborative network formed to include HIV laboratory services across Australia (Western Australia, South Australia, Victoria, Queensland and New South Wales: represented in this study by western Sydney). State ethics (Western Sydney Local Health District Human Research Ethics Committee, South Australian Human Research Ethics Committee for South Australia, Melbourne Health Human Research Ethics Committee, Pathology Queensland Institutional Review Board for Low and Negligible Risk Research and Royal Perth Hospital Ethics Committee), national ethics (University of Sydney Human Research Ethics Committee) and governance frameworks were established to include assessment of de-identified HIV-1 sequences where a unique identification number was assigned, along with the notification of each state, the year the sequence was performed, the gender and the age of the patient at the time of sequence. Data collection and analysis was performed on baseline HIV-1 plasma RNA samples analysed throughout Australia from 2005–2012 with the exception of Queensland where sequences from 2007–2012 were provided. In the case where multiple sequences for one patient were identified, the earliest sequence was determined to be the baseline sequence for the analysis. A total of 4,873 sequences were assessed and stratified over four time eras based on the year of sample collection (2005–2006, 2007–2008, 2009–2010 and 2011–2012).

### HIV-1 subtype determination

A contiguous *pol* sequence was generated spanning the protease (PR, amino acid positions 1–99 in HXB2) and reverse transcriptase region (RT, amino acid positions 20–240 in HXB2) sequences were amplified using in-house techniques (Western Australia [31], South Australia [32], Victoria [33], and western Sydney) or commercially available methods (Queensland; Viroseq HIV-1 Genotyping System, Abbott Celera). The HIV-1 subtype determinations were assigned by each state by submitting the fasta files to the calibrated population resistance tool (HIV db program) linked to the Stanford HIV database (http://hivdb.stanford.edu/) with the exception of Victoria who confirmed subtypes by submitting sequences to the Los Alamos database (http://www.hiv.lanl.gov) and the NCBI HIV genotyping tool (http://www.ncbi.nlm.nih.gov/projects/genotyping/).

All assays were monitored for quality according to the National Association of Testing Authorities accreditation standards and subjected to quality control procedures and the Royal College of Pathologists of Australasia (RCPA) or alternative quality assurance programs.

### HIV-1 phylogeny approach: Sequence alignments and processing

Data collation and analysis was performed on all de-identified samples at one site (Western Australia). This process involved utilising the BioEdit tool [43] for sequence alignment and assessing sequence quality, while the Molecular Evolutionary Genetics Analysis Version 6 (MEGA V6) phylogenetic tool [44], was used to construct phylogenetic trees and infer clustering patterns of similar sequences as previously described [39]. The sequences used in this study have been submitted into GenBank under the following accession identifiers: KY867758-KY872628.

### Characterisation of sequence similarities pertaining to clusters

For computational reasons the sequencing data was divided into B and non-B subtype analysis and in keeping with previous analyses [31–33,39] we removed drug resistance sites then defined clustering patterns of similar sequences according to the following conservative approach: (1) paired sequences where two sequences group together according to the criteria where the bootstrap (BS) value was ≥ 98% and the genetic distance (GD) between the two sequences was ≤ 1.5%; and (2) a “cluster or network” where three or more sequences had a BS value of ≥98% and a GD ≤1.5% from at least one other sequence in the cluster.

### Statistical analysis

Statistical analysis of the demographic data was performed using Statistical Package for the Social Sciences version 21.0 (SPSS v21: Armonk, NY: IBM Corp). Data distribution was assessed for normality then subjected to statistical analysis using T-tests, ANOVA and post hoc tests with correction for multiple comparisons in multivariable analyses. Results were considered statistically significant when *p*-values <0.05.

## Results

### Data distribution within the AMEN HIV-1 cohort

**Fig 1:**
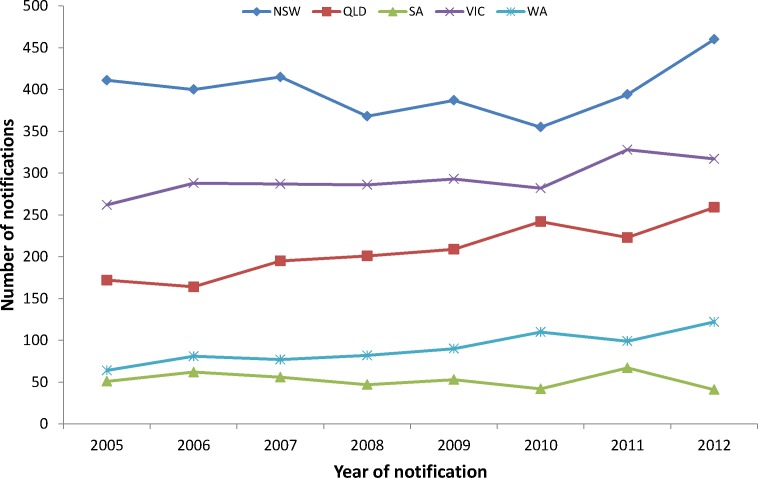
The number of new HIV-1 diagnoses by year for each Australian jurisdiction outlined in the Australian molecular epidemiology network; NSW = New South Wales, QLD = Queensland, SA = South Australia, VIC = Victoria and WA = Western Australia (Provided by the Kirby Institute [23]).

The underlying rates of HIV infection in Australia from 2005–2012 are shown for each state (Fig 1). Rates of HIV-1 infection are gradually increasing, overtime, for each state. The baseline HIV-1 sequence contribution from each Australian state from 2005–2012, and the corresponding subtype diversity for 4,873 sequences are shown in Table 1. Samples submitted by Victoria (n = 1,668, 34%) and Queensland (n = 1,579, 32%) account for 66% of the total samples for the study while Western Australia (15.5%), Western New South Wales (11%) and South Australia (7.5%) account for 44% of the sequences. Overall, 3631 (74.5%) HIV-1 infected individuals were infected with subtype B while 1,242 (25.5%) were infected with non-B viruses including A, C, D, F, G, CRF01_AE, CRF02_AG subtypes and other inter-subtype recombinant forms. Within the subtype B group there was a predominance of males (n = 4,203, 86%; Table 2), with evidence of non-B subtype virus in 19% of males (796/4,203) compared to 67% of females (436/648, *p*<0.0001). Females infected with a non-B subtype were on average six years younger than non-B infected males (32.5 years vs 38.5 years; *p*< 0.001) and 3.8 years younger than subtype B infected females (*p*<0.001). Females infected with subtype B were approximately three years younger than subtype B infected males (36.3 years vs 39.2 years; p<0.001).

**Table 1:** Contribution from each state toward the AMEN epidemiological and diversity study showing HIV subtype characteristics and baseline sequence information.

*State*	*Method*	*Year*		*Sequence (n)*	*SUBTYPE ID (assigned from Stanford DB)*							*Hospital/* *Service*
					B Subtype	CRF01_AE Subtype	C Subtype	CRF02_AG Subtype	D Subtype	Recombinant Forms	Other Subtypes	
WA	Stanford dB	2005–2012	724		427	139	109	17	5	23	4	RPH-DCI
SA	Stanford dB	2005–2012	351		240	38	35	20	1	9	8	SA Health
VIC	Los Alamos dB	2005–2012	1668		1261	174	153	10	4	49	17	VIDRL
W-Sydney	Stanford dB	2005–2011	551		407	43	54	21	3	13	10	Westmead
QLD	Stanford dB	2007–2012	1579		1296	90	124	20	8	34	7	Pathology QLD HIV Reference Lab
**Total**			4873		3631	484	475	88	21	128	46	

**Table 2:** Demographics for HIV-1 B subtype, non-B subtypes and sequences in a network including age, gender, AMEN centre and sequencing era.

Characteristic	All sequences	B subtype	Non-B subtype	Sequences in a network (%)	Sequences not in a network (%)
**All Sequences**	4873	3631	1242	1135 (23.1)	3738 (76.9)
**Gender**					
— **Female** — **Male** — **Other**	648 4203 22	212 3407 12	436 796 10	142 (21.9) 991 (23.6) 2 (13.0)	506 (78.1) 3212(76.4) 20 (87.0)
— **Age (Years)**	38.3	39	36.4	36.5	38.9
— **Male** — **Female** — **<18yo (n)** — **≥18yo (n)**	39.2 33.8 80 4773	39.2 36.3 23 3595	38.5 32.5 57 1178	39.6 31.7 26 1103	37.2 34.3 54 3645
**AMEN centre**					
— **WA** — **SA** — **VIC** — **WM NSW** — **QLD**	724 351 1668 551 1583	427 240 1261 407 1296	297 111 407 144 283	178 (24.6) 70 (19.9) 439 (26.3) 84 (15.2) 364 (23.1)	546 (75.4) 281 (80.1) 1229 (73.7) 467 (84.8) 1215 (76.9)
**Sequence Era**					
— **2005–2006** — **2007–2008** — **2009–2010** — **2011–2012**	809 1231 1424 1409	651 989 1016 975	158 242 408 434	119 (14.7) 256 (29.8) 359 (25.2) 401 (28.4)	690 (85.3) 975 (70.2) 1065 (74.8) 1008 (71.6)

### HIV-1 subtype diversity distributions from 2005–2012

**Fig 2:**
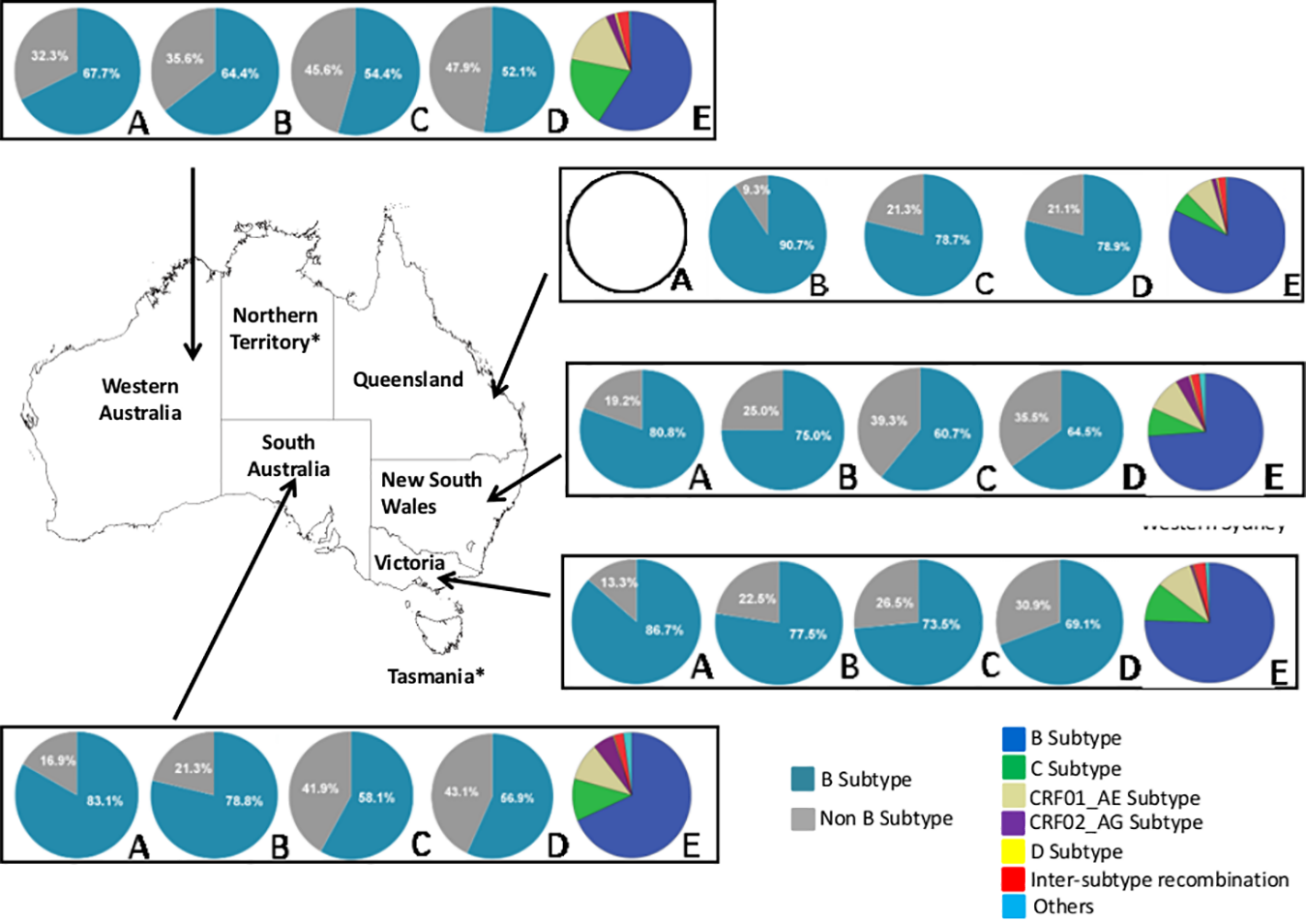
Australian HIV-1 subtype distribution for 4,873 sequences for B subtype (aqua) and non-B subtypes (grey) over time [2005–2006 (A), 2007–2008 (B), 2009–2010 (C) and 2011–2012 (D)] including the distribution and proportion of HIV-1 subtypes represented in each Australian state (E) from 2005–2012 (blue = B subtype; green = C subtype; tan = CRF01_AE subtype; maroon = CRF02_AG; yellow = D subtype; red = inter-subtype recombination (ISR); light blue = others).

As shown in Fig 2, a substantial increase in the proportion of HIV-1 non-B subtypes has been demonstrated within each state over time, with Western Australia having the highest proportion of baseline non-B sequences in the earliest time era (32.3% in 2005–2006), rising to approximately 48% in the period 2011–2012. Interestingly, the substantial increase in HIV-1 non-B subtypes observed in South Australia, western Sydney and Queensland occurred during 2009–2010 while Victoria showed a steady increase from 2007–2012.

Each state was represented by a range of HIV-1 subtypes including B, C, D, CRF01_AE, CRF02_AG subtypes and other inter-subtype recombinant forms (Fig 2). There were proportionally more subtype C (15.1%) and CRF01_AE (19.2%) sequences in Western Australia, more subtype B (82%) sequences in Queensland and more CRF02_AG (5.7%) sequences in South Australia than other states.

### Phylogenetic analysis

**Fig 3:**
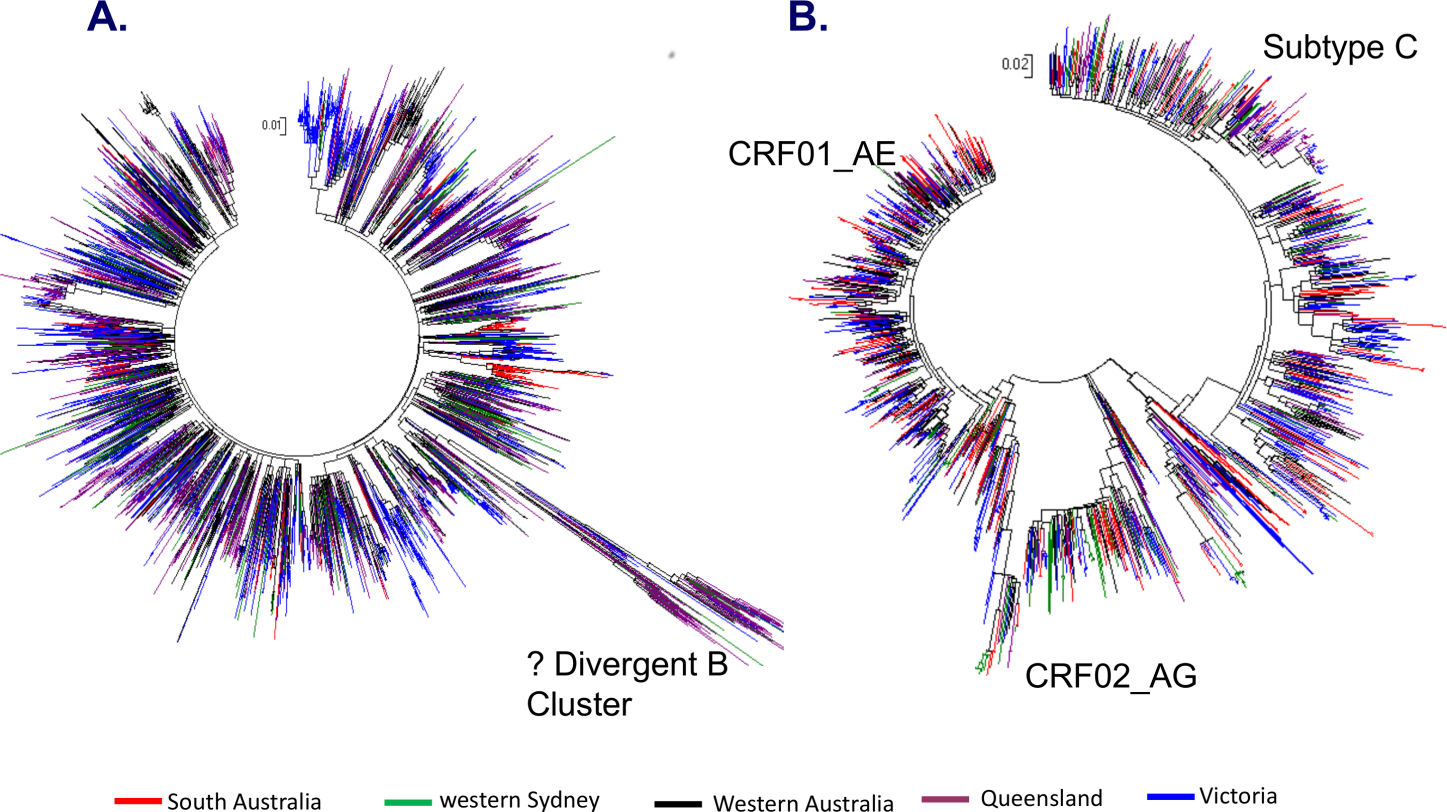
The phylogenetic tree constructions based on HIV-1 *pol* sequences for (A) HIV-1 B and (B) non-B subtypes assessed in the AMEN study. Each branch colour represents a state (Western Australia (black), South Australia (red), Victoria (blue), western Sydney (green) and Queensland (maroon).

The phylogenetic trees based on HIV-1 partial *pol* sequences of subtype B and non-B infections are represented in Fig 3A and 3B respectively. Overall, 1,135 of the 4,873 sequences (23%) were identified within a phylogenetic pair or cluster, with no difference between subtype B and non-B sequences (subtype B in a pair/cluster 829/3,631 (23%), non-B 306/1,242 (24.5%);*p* = 0.3). From the 419 pairs/clusters there were 286 paired sequences, 115 clusters with 3–5 sequences, 17 clusters with 6–13 sequences and one large cluster of 29 sequences.

**Fig 4:**
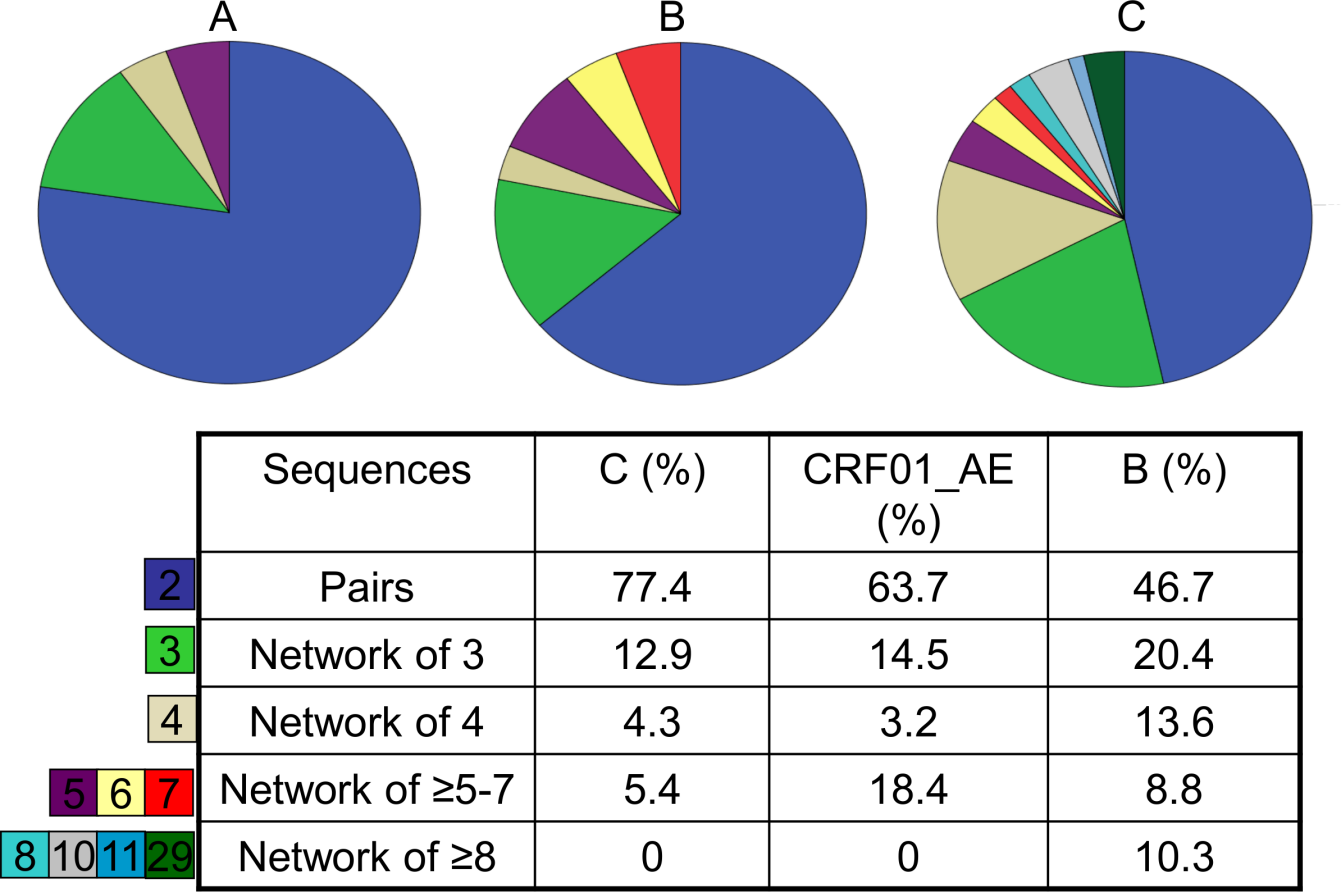
Differences in the distribution and proportions of pairs and larger cluster size for HIV-1 subtypes; HIV-1 C subtype (A), CRF01_AE subtype (B) and B subtype (C) (excludes singletons).

Analysing these results further in terms of subtype distribution, we found proportionally more potential transmission pairs within the subtype C cohort (77%; Fig 4A) compared to the CRF01_AE or subtype B cohorts (64%; Fig 4B and 47%; Fig 4C). There were also significantly more pairs in the non-B cohort than clusters (≥3 similar sequences: 94 vs 29, *p* = 0.003). The subtype B cohort had a significantly higher number of large clusters (≥4 sequences) compared with the non-B cohort (45% vs 24% *p* = 0.021).

**Fig 5:**
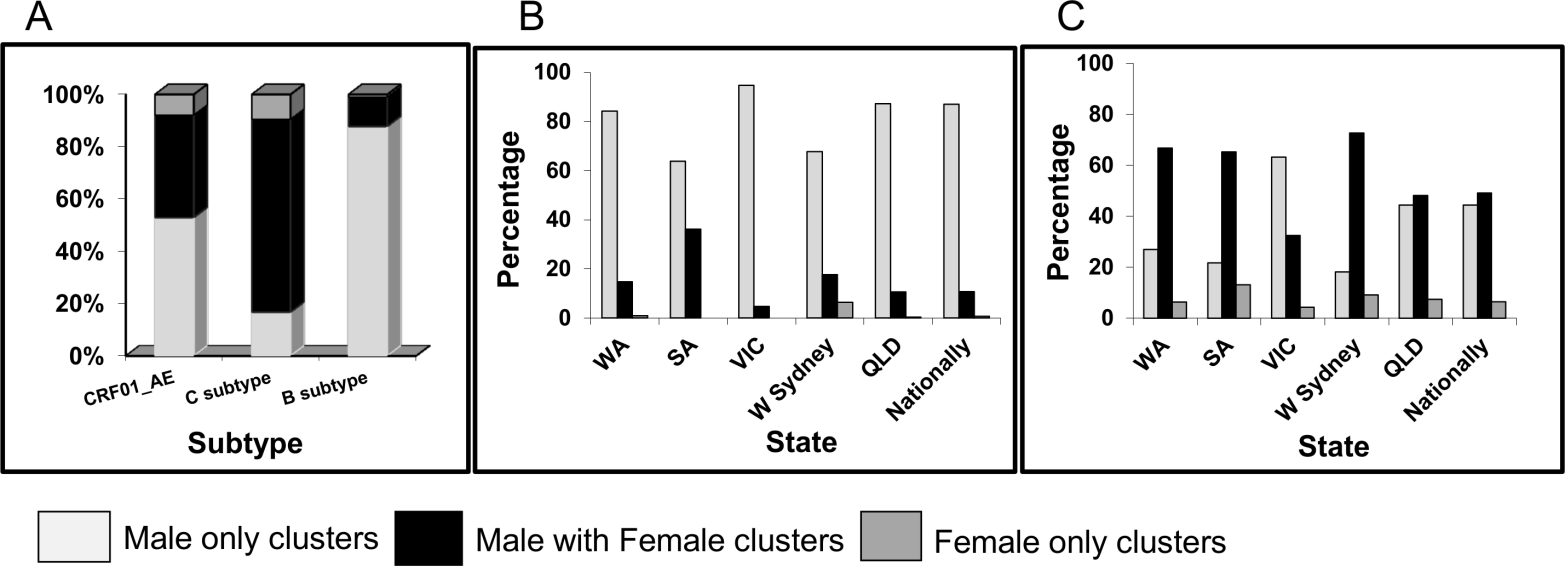
Gender distribution within clusters according to HIV-1 subtypes (A) and for clusters in each state with B subtype (B) or a non-B subtype (C); W Sydney = Western Sydney, QLD = Queensland, SA = South Australia, VIC = Victoria and WA = Western Australia.

At a national level, the gender distribution within clusters were remarkably different between subtype B, C and CRF01_AE (Fig 5A, Anova: *p*<0.001). As expected a higher proportion of subtype B pairs and clusters were male-only (88%) compared to CRF01_AE and subtype C clusters (53% and 17%, respectively). Significantly more subtype C pairs and clusters included males and females (74%) compared to CRF01_AE (39%) and subtype B (11%).

The phylogenetic analysis of the subtype B and non-B cohorts was further assessed state by state. Within the subtype B cohort (Fig 5B), most states had high proportions of male-only clusters (>84%), with Victoria registering the highest proportion (95%). The two exceptions were South Australia and western Sydney, with lower proportions of male-only subtype B pairs/clusters (64% and 68% respectively) and correspondingly higher proportions of male-female pairs/clusters (36% and 18%, respectively).

When assessing non-B cluster dynamics, Victoria had the largest proportion of male-only non-B clusters (63%) followed by Queensland (44%) (Fig 5C). Conversely, western Sydney, Western Australia and South Australia experienced the highest proportion of non-B male/female pairs/clusters (73%, 67% and 65% respectively) followed by Queensland and Victoria (49% and 32%, respectively).

### Analysis of large sub-epidemic HIV-1 clusters

In the large sub-epidemic cluster analysis we found five distinct clusters with more than nine patient sequences with closely related sequences represented per cluster. The largest cluster, comprising subtype B, was initially identified in 2008 by three related sequences, increasing to include 29 sequences by 2012. All sequences were from males, including 27 sequences from Western Australia and two from Victoria, with a median age of 47.5 years (range 23–70). The overall mean genetic difference for this cluster was 0.3% (range, 0.2–1.2%) with a bootstrap value of 99%. Other subtype B clusters included two comprising of 10 sequences, one comprising of 11 sequences (these three clusters were all males originating from Victoria) and one subtype B male/female cluster of ten sequences which included sequences from three states (Western Australia, South Australia and Victoria), with 80% of the sequences notified in South Australia.

### Factors influencing large HIV-1 networks of similar sequences

We employed a multivariate regression analysis to show factors associated with being in a transmission pair or cluster, including factors associated with being in a large cluster (≥4 sequences). Both analyses excluded children <18 years of age.

**Fig 6:**
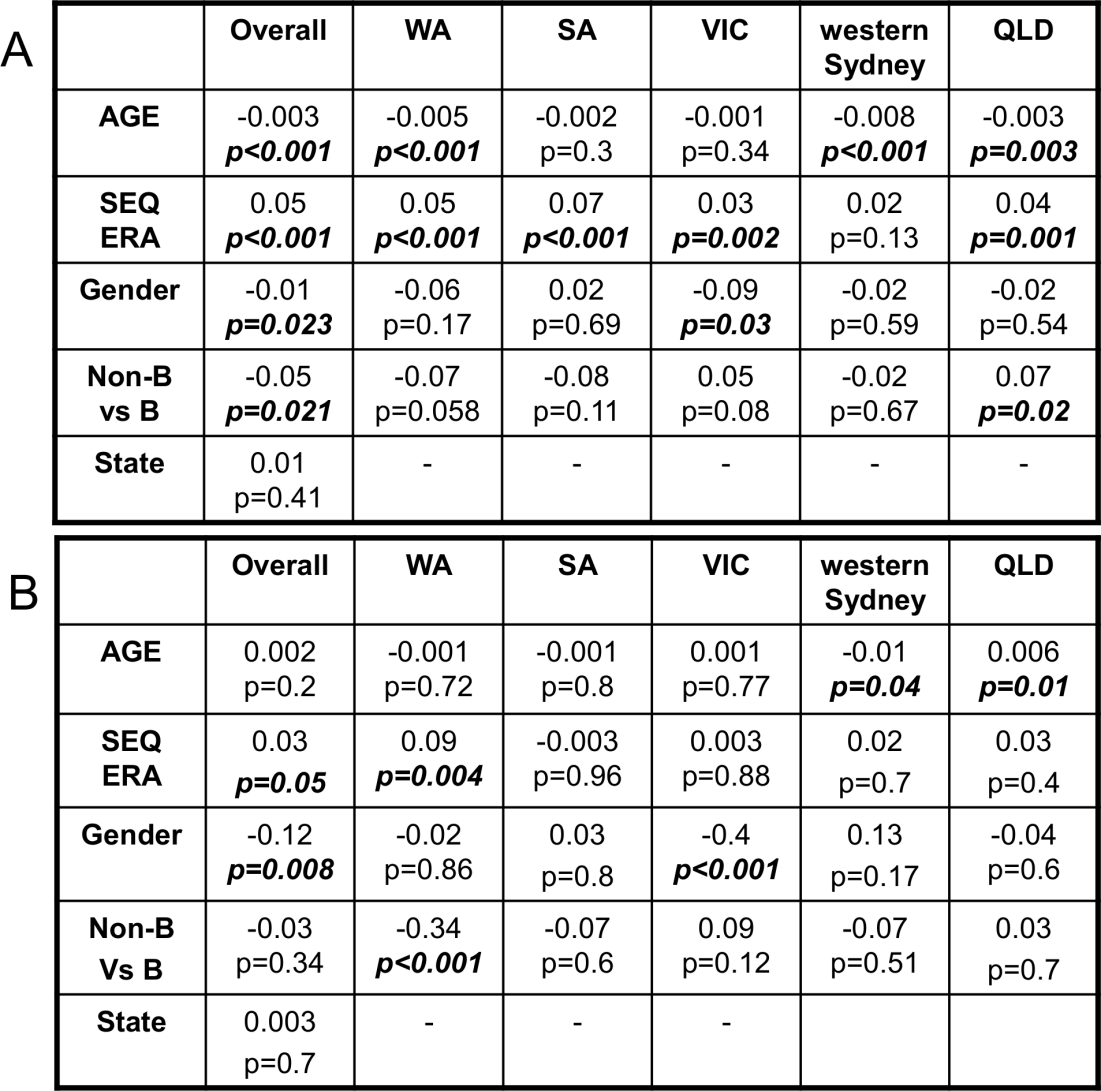
Multivariate regression analysis reveals factors associated with a sequence being classified into (A) a cluster regardless of cluster size or as a singleton or (B) only sequences in larger networks where the sequence size equals 2–3 or ≥4. All results are for >18yo (Coefficient and significance are shown); QLD = Queensland, SA = South Australia, VIC = Victoria and WA = Western Australia.

Factors associated with being part of a cluster (regardless of size) are presented in Fig 6A. At a national level associations were found with younger age (β -0.003; *p*<0.001), having a sequence performed during later era (β 0.05; *p*<0.001), being male (β -0.01; *p* = 0.023) and having a subtype B infection (β -0.05; *p* = 0.021). No significant association with the state the sequence originated from was found (β 0.01 *p* = 0.4). Differences within each state revealed a younger age was associated with being in a cluster for Western Australia, western Sydney and Queensland, while more recent HIV-1 sequencing was a significant factor for being in a cluster for all states besides western Sydney. Victorian males were significantly more likely to be in a cluster while non-B sequences were associated with transmission pairs or small clusters (3 sequences) in Queensland.

When factors associated with being in a large cluster (≥4 sequences) were compared with pairs or clusters of three (Fig 6B), there was a weak association with having a sequence performed during a later era (β 0.03; *p* = 0.05) and a strong association with being male (β 0.12; *p* = 0.008). In Western Australia, having a subtype B infection (β -0.34; *p*<0.001) and being sequenced in the current era (β 0.09; *p* = 0.004) were associated with larger cluster size. Younger age was associated with large cluster size in western Sydney (β -0.01; *p* = 0.04) while older age was identified as an association in Queensland (β 0.006; *p* = 0.01). Male gender was a significant factor in Victoria (β 0.4; *p*<0.001).

### Phylogenetic clusters within and between Australian states

**Fig 7:**
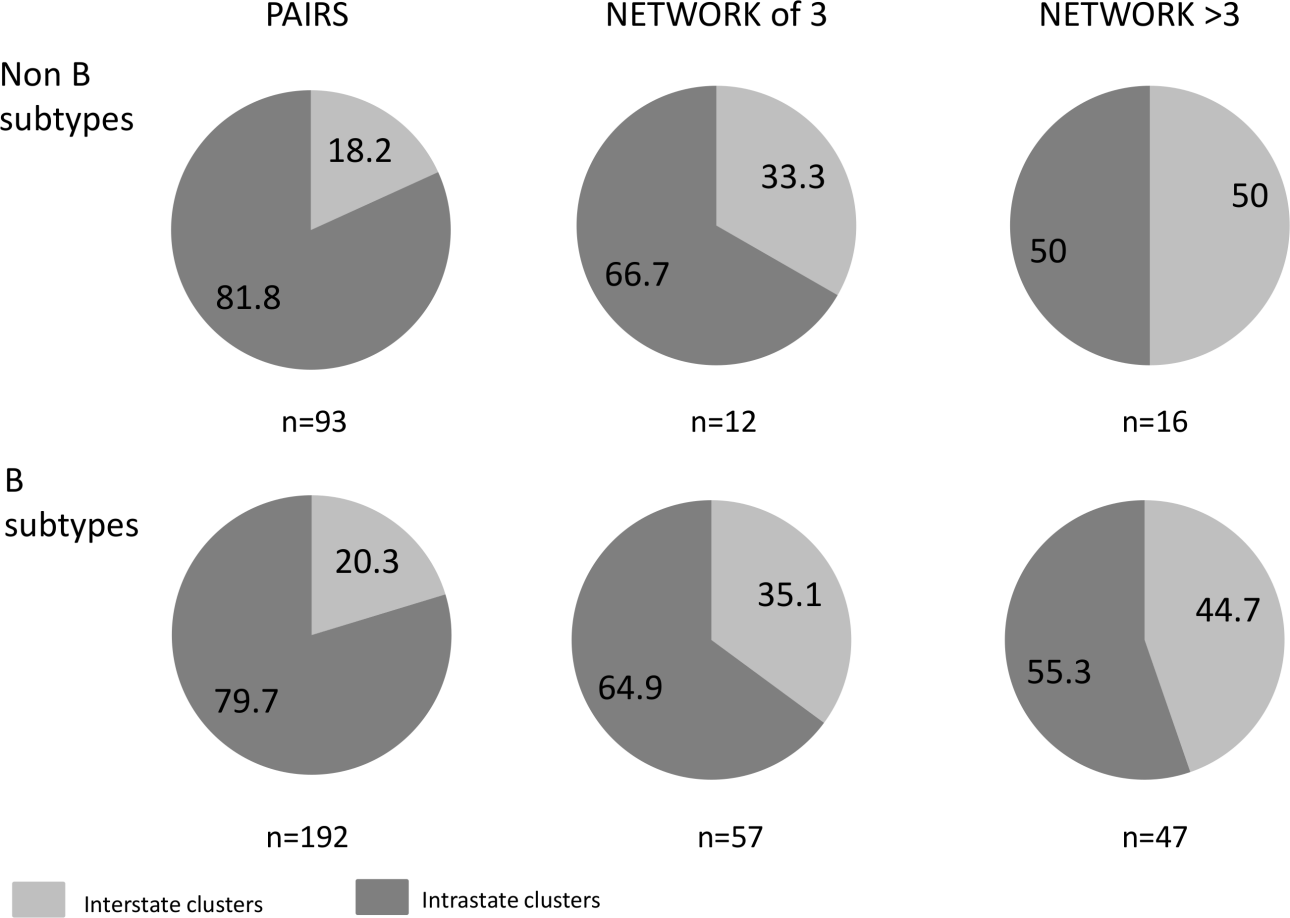
The proportion of interstate and intrastate HIV-1 paired sequences, sequences in a network of 3 or networks > 3 (n = # of pairs or networks).

Monitoring HIV-1 sequence similarities within (intrastate) and across state boundaries (interstate) was assessed for both subtype B and non-B cohorts (Fig 7), including only the first sequence derived from an individual patient who contributed sequence data in more than one Australian state. There were similar proportions of B and non-B HIV phylogenetic clusters that included sequences from more than one Australian state, increasing from 19.6% (56/285) of paired clusters (20.3% of subtype B pairs versus 18.2% of non-B pairs) to 46.0% (29/63) of clusters comprising >3 sequences (44.7% subtype B clusters >3 in size versus 50% of non-B clusters).

## Discussion

The outcomes of this study highlight an Australian HIV-1 epidemic characterised by an increasing prevalence of non-B infections with an overall expanding subtype diversity. Just over one quarter of all infections were identified as non-B subtypes, which is slightly lower than observed in recent studies undertaken in North America [4], Europe [6] and Belgium [45]. The dominant non-B subtypes identified in Australia are subtype C and CRF01_AE, the main HIV-1 strains circulating amongst sub-Saharan African and south-east Asian populations. A recent report has documented that the increase in CRF01_AE infections into previously low prevalence countries can be attributed to travel from Asian countries [35,36], with Thailand, Malaysia, China and India recently identified in the top 10 countries where an increase in Australian visitors has been recorded [28]. Phylogenetic analysis found that these non-B infections are more likely to occur within heterosexual transmission networks. The similar proportion of males and females infected with subtype C in Australia is consistent with the distribution of heterosexual transmissions documented in sub-Saharan Africa [46] while the increasing proportion of CRF01_AE infections in Australia amongst both females and males corresponds with south-east Asian patterns of heterosexual transmission and injecting drug use (IDU), as well as increasing MSM transmissions and injecting drug use (IDU) present in south-east Asia [35,47].

This study suggests that there is a growing contribution of migration and travel, both within Australia and overseas, to the increased HIV-1 subtype diversity nationally and within each of the Australian states. This is consistent with evidence of sharp increases in HIV-1 subtype diversity across the United States [4,38] and Europe [3,39–41]. Previous investigations have also recognised the evolution of HIV-1 diversity within Australia, from an early epidemic predominantly characterised by subtype B infection [25] to recent studies showing increasing HIV-1 non-B subtype diversity [31–33].

HIV-1 phylogenetic analysis provides an important objective resource for dynamically assessing new HIV infections and monitoring geographical changes in the global HIV epidemic. In this study, in which we classified phylogenetic clusters using a conservative approach that required low genetic distance between sequences (≤1.5%) as well as high bootstrap values (>98%), distinct transmission network patterns for subtype B, C and CRF01_AE were revealed. This approach has been successfully utilised to monitor both B [6,34,45] and non-B subtype [34,39,45] cluster patterns, however no definite consensus has been established to define clusters. Further sensitivity studies are therefore warranted and could be achieved by including multiple sequences from the same individual, developing criteria from known transmission pairs/networks and from sequencing a second HIV-1 gene.

As expected, the majority of pairs, small clusters and larger networks (≥4 sequences) were identified within male-only groups in the subtype B infected cohort, while there was a higher proportion of male-and-female clusters in the subtype C infected cohort compared with CRF01_AE. Evidence of emerging male-only non-B networks in Victoria suggests likely forward transmission of these viral subtypes within local MSM transmission networks, while the large proportion of male-female pairs within non-B transmission networks in western Sydney, South Australia and Western Australia suggest limited forward transmission into local networks.

The observation of a large subtype B male network, identified in 2008 and crossing state borders to evolve into a network of 29 patients by 2012, is in line with other epidemiological studies [6,45] showing that the size of transmission networks is not normally distributed and tends to involve one large cluster as well as a range of smaller networks. This requires further investigation that acknowledges both the social and behavioural context of HIV-1 transmission as well as the potentially important role of HIV-1 virulence, given a recent US study showed large cluster size was associated with CD4^+^ T cell counts >350 cells/μl and plasma HIV-1 RNA levels >10,000 copies/mL [38]. This observation is also supported by a recent study within Western Australia [31], and can be considered within a broader context of evidence that HIV-1 has adapted over time to evade host immunological control [48], with trends towards higher pre-treatment plasma HIV RNA levels over the past 30 years of the HIV epidemic [49].

Understanding the increase in HIV-1 diversity within Australia is of significant interest to laboratories as inaccurate HIV-1 RNA quantitation of non-B subtypes, mostly due to genetic variation at primer sites, has previously been reported [22]. It is therefore imperative that the evaluation of HIV-1 RNA assays be conducted in light of existing Australian HIV-1 viral diversity, to ensure reliable monitoring of HIV disease and treatment. Increased drug resistance has also been shown in non-B subtypes when using integrase inhibitors [50], while concerns regarding the potential failure of NNRTI/NRTI treatment for those infected with subtype C infection has been described [19]. The accurate determination of antiretroviral drug resistance mutations in light of HIV-1 subtype-associated polymorphisms also requires careful consideration in the context of increasing non-B subtype diversity [51]. Subtype D infection, ISRs [15,16] and CXCR4 tropism [18] associated with CRF01_AE [52] have all been associated with rapid disease progression, while subtype A has been associated with slower disease progression [53]. These factors may be important for HIV surveillance and patient care in developed countries.

To our knowledge this analysis is the first large-scale assessment of HIV-1 subtypes and phylogenetic network patterns in Australia performed over an eight-year period. While this current investigation provides estimates of this HIV-1 genotyping in Australia it does not provide a link between the distribution and evolution data with human behaviour, including high-risk populations. Future investigations are warranted and should be focused on filling this gap as has been achieved in Asia [35].

The study does highlight the key influences of migration and overseas travel on increasing rates of non-B infections, and reinforces the importance of Australia’s engagement with regional and global aspects of the HIV epidemic as well as the importance of engagement between Australian state jurisdictions. In this context, we hope that the establishment of the Australian Molecular Epidemiology Network, and the results from this first national study provide the basis for a greater understanding of the Australian HIV-1 epidemic, and enhances existing national surveillance methods. This information assists in the development of effective laboratory strategies, as well as informing prevention and treatment strategies to influence the HIV epidemic within Australia and beyond.
